# Degenerated, Undifferentiated, Rearranged, Lost: High Variability of Sex Chromosomes in Geometridae (Lepidoptera) Identified by Sex Chromatin

**DOI:** 10.3390/cells10092230

**Published:** 2021-08-28

**Authors:** Martina Hejníčková, Martina Dalíková, Pavel Potocký, Toomas Tammaru, Marharyta Trehubenko, Svatava Kubíčková, František Marec, Magda Zrzavá

**Affiliations:** 1 Faculty of Science, University of South Bohemia, Branišovská 1760, 370 05 České Budějovice, Czech Republic; martina.hejnickova@entu.cas.cz (M.H.); dalikm00@prf.jcu.cz (M.D.); trehum01@prf.jcu.cz (M.T.); 2 Biology Centre of the Czech Academy of Sciences, Institute of Entomology, Branišovská 31, 370 05 České Budějovice, Czech Republic; p.potocky@entu.cas.cz (P.P.); marec@entu.cas.cz (F.M.); 3 Institute of Ecology and Earth Sciences, University of Tartu, Vanemuise 46, 51014 Tartu, Estonia; toomas.tammaru@ut.ee; 4 Veterinary Research Institute, Hudcova 70, 621 00 Brno, Czech Republic; kubickova@vri.cz

**Keywords:** sex chromosome evolution, W chromosome, neo-sex chromosomes, sex chromatin, Lepidoptera, Geometridae, comparative genomic hybridization, intraspecific chromosomal variability

## Abstract

Sex chromatin is a conspicuous body that occurs in polyploid nuclei of most lepidopteran females and consists of numerous copies of the W sex chromosome. It is also a cytogenetic tool used to rapidly assess the W chromosome presence in Lepidoptera. However, certain chromosomal features could disrupt the formation of sex chromatin and lead to the false conclusion that the W chromosome is absent in the respective species. Here we tested the sex chromatin presence in 50 species of Geometridae. In eight selected species with either missing, atypical, or normal sex chromatin patterns, we performed a detailed karyotype analysis by means of comparative genomic hybridization (CGH) and fluorescence in situ hybridization (FISH). The results showed a high diversity of W chromosomes and clarified the reasons for atypical sex chromatin, including the absence or poor differentiation of W, rearrangements leading to the neo-W emergence, possible association with the nucleolus, and the existence of multiple W chromosomes. In two species, we detected intraspecific variability in the sex chromatin status and sex chromosome constitution. We show that the sex chromatin is not a sufficient marker of the W chromosome presence, but it may be an excellent tool to pinpoint species with atypical sex chromosomes.

## 1. Introduction

Sex chromosomes represent a rapidly evolving part of the genome. It is generally accepted that they originate from a pair of autosomes when one of the homologs has acquired a sex-determining factor [1]. This triggers a sequence of events conditioned by the cessation of recombination, leading to the degeneration of a sex-specific sex chromosome, i.e., Y or W [2]. Typical features of the Y and W chromosomes are gene deficiency, the presence of pseudogenes, and the abundance of repetitive sequences such as mobile elements and tandem repeats. Eventually, the sex-specific chromosome may lose its sex-determining function and disappear, resulting in the X/XX or Z/ZZ sex chromosome system (reviewed, e.g., in the work of [3,4]). Alternatively, new heteromorphic sex chromosomes may arise from a B chromosome, which acquires a sex-determining locus or simply begins to pair with the X or Z chromosome in the heterogametic sex [5,6].

The evolution of sex chromosomes, however, may not be so direct. Apart from the canonical XX/XY or WZ/ZZ systems, neo-sex chromosomes may arise by fusion between the ancestral sex chromosomes and autosomes. These neo-sex chromosomes then consist of multiple evolutionary strata, which may be clearly visible on the neo-Y or neo-W chromosome of some species due to the heterochromatin part of the ancestral degenerate sex chromosome and the euchromatin part of the attached autosome (e.g., the work of [7,8,9]). In addition, multiple sex chromosome systems may arise by fissions of ancestral sex chromosomes or by fusions and translocations between sex chromosomes and autosomes (e.g., the work of [10,11]).

The evolution of sex chromosomes thus proceeds in cycles: from non-degenerate, autosome-like sex chromosomes, through various stages of ongoing degeneration of the Y and W chromosomes, to their ultimate loss, while taking detours through neo-sex chromosomes in the meantime. Therefore, differentiated sex chromosomes in different species can be achieved at various stages of the process, and their number, appearance, and molecular content can vary enormously, even among related species [12,13,14]).

Moths and butterflies (Lepidoptera) are the most species-rich group with female heterogamety. Diverse sex chromosome systems have been described in this group, including primary absence or secondary loss of the W chromosome, neo-sex chromosomes, and multiple sex chromosomes, allowing to study the sex chromosomes at various stages of their evolution. Lepidoptera, therefore, represent an ideal model for the study of sex chromosome evolution. The basal clades of the order Lepidoptera and the sister order Trichoptera (caddisflies) lack the W chromosome, the absence of which is thus considered an ancestral state [15,16]. The exact mechanism and time of the W chromosome origin are not yet clear [17], but it is typically present in Ditrysia, a megadiverse group comprising 98% known lepidopteran species. Accordingly, the most common sex chromosome constitution in this group is WZ/ZZ (♀/♂) [15].

The lepidopteran Z chromosome resembles autosomes in content and structure [18]. It is rich in genes and exhibits a highly conserved synteny block of “ancestral” Z-linked genes across Lepidoptera [17,19,20,21]. In contrast, most of the W chromosomes investigated so far consist mainly of repetitive sequences and probably contain very few functional genes [22,23]. A high load of repeats leads to the conversion of the W chromosome to heterochromatin, which is often the largest or only heterochromatin block in the karyotype. As a consequence, the W chromosome of most species forms a round condensed body in female interphase polyploid nuclei, the sex chromatin, which contains up to several thousand W copies [22].

In a comprehensive study monitoring the occurrence of sex chromatin in 238 species, Traut and Marec [24] concluded that sex chromatin is a common trait of females in Ditrysia. They also emphasized the usefulness of sex chromatin as a diagnostic marker of sex in young developmental stages and a marker for the identification of W chromosome aberrations. Since then, sex chromatin has been used in a number of other studies as an indicator of the W chromosome presence and its condition (e.g., the work of [7,17,25,26,27]). However, the use of sex chromatin as a marker should be considered with caution, as there are certain cytogenetic factors that might influence its occurrence and appearance. For example, the fusion of a W chromosome with a Z or an autosome or translocation from another chromosome onto a W chromosome may result in fragmentation, an aberrant shape, or even disappearance of the sex chromatin body. This has been observed in irradiated strains of the Mediterranean flour moth (*Ephestia kuehniella*) [18,28,29] or the codling moth (*Cydia pomonella*) [30], but also in species in which the fusion of W with another chromosome occurred naturally, such as the vapourer moth (*Orgyia antiqua*), or the clouded Apollo (*Parnassius mnemosyne*) [9,31].

Sex chromatin, if present, represents an excellent source of the W chromosome DNA that can be used either to prepare a W-painting probe for W chromosome identification by fluorescence in situ hybridization (FISH) [13,17,32,33] or for sequencing [23]. Another technique used to study W chromosomes is comparative genomic hybridization (CGH), in which differently labeled male and female genomic probes compete on complementary loci on chromosomes of heterogametic sex [34,35]. CGH thus not only identifies the W chromosome but also provides information about its sequence composition. This method has, however, its limitations as it fails to detect molecularly undifferentiated sex chromosomes [36].

In this study, we investigated the W chromosomes in geometrid moths. Geometrids are one of the most diverse groups of Ditrysia, with over 23,000 species [37]. Due to their advanced phylogenetic position, the presence of sex chromatin and W chromosome is expected. However, available studies show an unusual occurrence of sex chromatin in this group. Of the 23 species tested so far, the sex chromatin pattern was atypical in females in 7 of them, despite the fact that the species were phylogenetically distant ([38,39]; reviewed in the work of [24]). Furthermore, geometrids differ noticeably in chromosome numbers, suggesting dynamic karyotype evolution by fusions and fissions that could include sex chromosomes [40,41]. Yet, very little has been done regarding cytogenetic analysis of geometrid sex chromosomes [13,20]. Therefore, we conducted an extensive survey of the sex chromatin status in geometrids, which included 50 species. Based on the results, we selected eight species with atypical and normal sex chromatin patterns and performed a detailed cytogenetic analysis to identify the cause of sex chromatin malformation or absence. We also addressed questions on the use of sex chromatin as a W chromosome marker and as a data source for theories on the W chromosome origin (e.g., the work of [15,17,25]).

## 2. Materials and Methods

### 2.1. Specimens

The list of 50 species of Geometridae screened for the presence of sex chromatin in adulthood is included in Table S1. Based on the results, 8 species were chosen for thorough cytogenetic investigation: *Aethalura punctulata*, *Chiasmia clathrata*, *Epirrhoe alternata*, *Hylaea fasciaria*, *Hypomecis atomaria*, *Operophtera brumata*, *Peribatodes rhomboidaria*, and *Pseudopanthera macularia*. Except for *E. alternata* and *O. brumata* (Larentiinae), all species belong to the subfamily Ennominae [42]. Adult moths were collected in Czechia and Estonia in the years 2018–2020, either using UV light traps at night or captured by entomological nets in daylight; brachypterous females were collected from tree trunks at night. All specimens were identified according to their morphology. Mated females were kept individually in moisturized plastic containers with host plants, where they laid eggs. The hatched larvae were reared on their host plants until the last instar or pupa, dissected, and the residual tissue was immediately frozen in liquid nitrogen and stored at −20 °C. From each selected species, we examined several offspring of both sexes from different mothers and from different localities (for more details, see Table S2).

### 2.2. Chromosomal Preparations and Sex Chromatin Assay

Chromosomal preparations were obtained from penultimate instar larvae from gonads (meiotic chromosomes) and from wing imaginal disc or brains (mitotic chromosomes) as described in the work of [27]. Polyploid interphase nuclei for sex chromatin assay were obtained from Malpighian tubules of both larvae and adult specimens and stained with lactic acetic orcein as described in the work of [43].

### 2.3. DNA Isolation

Genomic DNA (gDNA) was isolated by CTAB (hexadecyltrimethylammonium bromide; Sigma-Aldrich, St. Louis, MO, USA) according to the protocol of [44] with the following modifications. Insect tissues were crushed in 800 µL of extraction buffer prepared according to the protocol and incubated in a heat block at 60 °C overnight. Afterward, an equal amount of pure chloroform was added, and the sample was centrifuged, the upper aqueous phase was transferred into a new tube, and the chloroform extraction step was repeated. The solution was then treated with 5 µL of RNase A (10 mg/mL; Sigma-Aldrich) and incubated for 30 min at 37 °C. To precipitate DNA, 2/3 of the final volume of isopropyl alcohol (Sigma-Aldrich) was added, and the mixture was incubated for at least 2 h at room temperature. Finally, the solution was centrifuged for 15 min at 14,000 g, the supernatant was removed, and the pellet was washed in 70% ethanol, air-dried, and dissolved in PCR-grade water. Final concentrations of the extracted gDNA were measured on a Qubit 3.0 fluorometer (Invitrogen, Carlsbad, CA, USA), and DNA purity was assessed by 260/280 ratio on a Nanodrop 2000 spectrophotometer (Thermo Fisher Scientific, Waltham, MA, USA).

### 2.4. Comparative Genomic Hybridization (CGH)

Genomic DNAs were fluorescently labeled by the improved nick translation procedure of [45] with some modifications [27]. Male gDNAs were labeled with Cy3-dUTP, female gDNAs with either fluorescein-dUTP or ATTO 488-dUTP (all Jena Bioscience, Jena, Germany). Nick translation reactions were incubated at 15 °C and stopped after 3.5 h either by 10% of the reaction volume loading dye buffer (25 mM EDTA pH 8, 0.6 mM bromophenol blue, and 5% glycerol) or by 10 min inactivation at 70 °C. Labeled probes were checked on a standard 1.5% agarose gel in TAE buffer.

CGH was carried out according to the protocol of [34] with modifications described in the work of [17]. Briefly, the hybridization mixture per slide consisted of 250–500 ng of each labeled gDNA probe (exactly the same amount of gDNA of each sex per slide) and 25 µg of sonicated salmon sperm DNA (Sigma-Aldrich) as a non-specific competitor. The mixture was precipitated and dissolved in 50% deionized formamide, 10% dextran sulfate, and 2 × SSC. After 5 min incubation at 90 °C, it was cooled down on ice and prehybridized for 1.5 h at 37 °C. Finally, the mixture was applied on a slide, which had already been incubated with RNase A (200 ng/µL, Sigma-Aldrich) in 2 × SSC for 1 h at 37 °C, twice washed in 2 × SCC for 5 min, and denatured with 70% formamide in 2 × SSC at 68 °C for 3.5 min, then cooled down in 70% cold ethanol (1 min) and dehydrated in 80% and 100% ethanol at room temperature, 30 s each. Slides were incubated with the hybridization mixture at 37 °C for 3 nights. They were then washed for 5 min at 62 °C in 0.1 × SSC with 1% Triton X-100, stained with 0.5 µg/mL DAPI (4′,6-diamidino-2-phenylindole; Sigma-Aldrich), and mounted in DABCO antifade (1,4-diazabicyclo(2.2.2)-octane; Sigma-Aldrich). In each species studied, several specimens of both sexes were examined by CGH.

### 2.5. Genomic In Situ Hybridization (GISH) with 18S rDNA Probe

GISH was performed according to the CGH protocol (see above), only without the prehybridization step. The hybridization mixture consisted of 250–500 ng of a female gDNA probe labeled by nick translation (see above), 3 µg of unlabeled male gDNA (fragmented by boiling at 99 °C for 20 min), 25 µg of sonicated salmon sperm DNA, and 300 ng of labeled 18S rDNA probe. This probe was prepared by PCR from the codling moth, *Cydia pomonella*, following the protocol described in the work of [46] and labeled with Cy3-dUTP by nick translation with 1.5 h incubation.

### 2.6. Reprobing and Fluorescence In Situ Hybridization (FISH) with Telomeric Probe

In species with supposed sex chromosome multivalents, we performed FISH with a telomeric probe on slides previously used for CGH to visualize chromosome ends. The insect telomeric probe (TTAGG)*_n_* [47] was generated by non-template PCR [48] as described in the work of [49] and labeled with biotin-14-dUTP (Invitrogen) by nick translation (see above) with 50 min incubation. Genomic probes were removed from slides as described in the work of [50]. The telomeric probe was then hybridized on slides followed by multiple signal amplification steps, using streptavidin-Cy3 conjugated antibodies (Jackson ImmunoRes. Labs., Inc., West Grove, PA, USA) and biotinylated antistreptavidin antibodies (Vector Labs., Inc., Burlingame, CA, USA) according to the protocol described in the work of [49]. Finally, the slides were counterstained with 0.5 µg/mL DAPI in DABCO antifade.

### 2.7. FISH with W-Painting Probe

In *C. clathrata* and *O. brumata*, we microdissected sex chromatin and used it as a painting probe to visualize the W chromosome. Microdissection was performed essentially as described in the work of [32], and probe amplification and labeling were performed as described in the work of [17]. In *C. clathrata*, the W-painting probe labeled with Cy3-dUTP was hybridized along with the telomeric probe labeled with ATTO 488-dUTP as described in the work of [17] with the following modifications: the amount of telomeric probe was 300 ng per slide, and post-hybridization washes were 2 × 5 min in 2 × SSC, 2 × 5 min in 1 × SSC, 1 min in 1 × PBS and 1 min in 1% Kodak Photo-Flo in miliQ water, all at 25 °C. These low stringency washes were used due to the low intensity of hybridization signal of the W-painting probe, observed after the high stringency wash in 0.1 × SSC with 1% Triton at 62 °C for 5 min, conditions that we otherwise use for FISH with fluorochrome-labeled probes. In *O. brumata*, the W-painting probe labeled with Cy3-dUTP was used without the telomeric probe, and the high stringency post-hybridization wash was applied.

### 2.8. Microscopy and Image Processing

Chromosomal preparations were examined under a Zeiss Axioplan 2 microscope (Carl Zeiss, Jena, Germany). For fluorescently stained chromosomes, we used narrow fluorescence filter sets and a monochrome CCD camera XM10 (Olympus Europa Holding, Hamburg, Germany). Polyploid nuclei preparations stained with lactic acetic orcein were examined by light microscopy using 40x and 63x objectives depending on the size of the nuclei. Black-and-white images were captured by cellSens Standard software version 1.9 (Olympus). For slides after CGH, GISH, or FISH, pictures were captured separately for each fluorescent dye, then pseudocoloured and merged using Adobe Photoshop CS6 (Adobe Systems, San Jose, CA, USA).

## 3. Results

**Figure 1:**
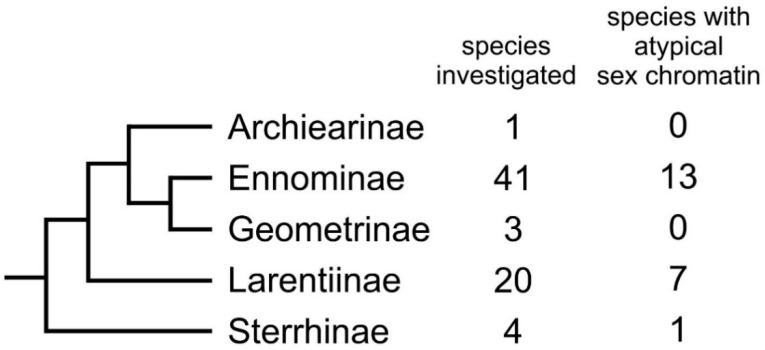
Species inspected for the sex chromatin presence. Data on 69 species of Geometridae belonging to five subfamilies are included. An atypical sex chromatin pattern was found in almost one-third of the species (see Table S1 for details). Phylogeny based on the work of [42].

We inspected 50 species from five subfamilies of Geometridae for the presence of a sex chromatin body in polyploid nuclei of the Malpighian tubule cells. Including data from the literature, the female sex chromatin assay in 69 species revealed several variants: normal sex chromatin (i.e., the usual conspicuous body) was present in 48 species, while atypical sex chromatin (i.e., missing, scattered, miniature, multiple or variable) was found in 21 species (Figure 1; Table S1). Males of all inspected species lacked sex chromatin.

Based on the sex chromatin assay, we selected six species with atypical sex chromatin as well as two species with normal sex chromatin as controls for a thorough cytogenetic investigation (see Table 1 for a summary of results).

### 3.1. Aethalura Punctulata (Ennominae)

**Figure 2:**
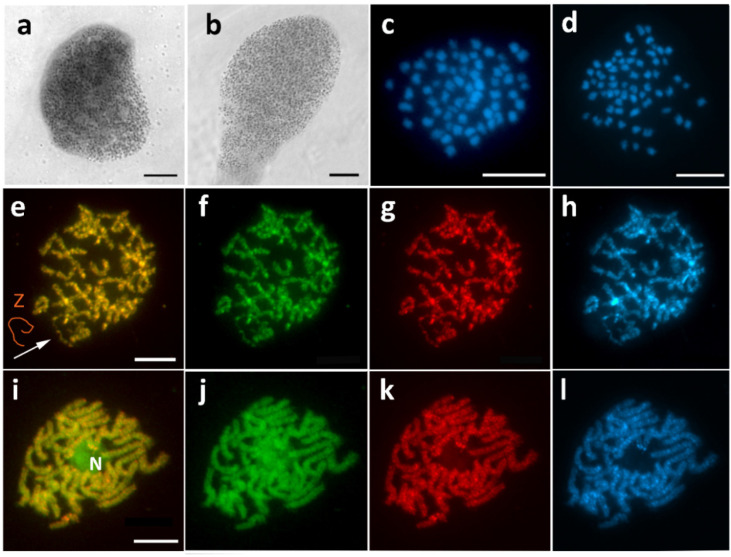
Missing W chromosome in *Aethalura punctulata*. Sex chromatin was absent in female (**a**) and male (**b**) polyploid nuclei stained with orcein. Mitotic metaphase chromosomes stained with DAPI show 2*n* = 61 in females (**c**) and 2*n* = 62 in males (**d**). Comparative genomic hybridization (CGH) on female pachytene chromosomes (**e**–**h**) did not reveal any differentiated chromosome. The Z univalent is indicated by an arrow and shown in the scheme (**e**). CGH on male pachytene chromosomes showed similar results, but no univalent was found (**i**–**l**); note nucleolus (N). Panels (**e**,**i**)—merged pictures of both probes; (**f**,**j**)—female genomic probe (green); (**g**,**k**)—male genomic probe (red); (**h**,**l**)—DAPI staining (light blue). Bar = 10 µm.

Sex chromatin was completely missing in *A. punctulata* (Figure 2a,b). Subsequently, the absence of the W chromosome was confirmed by chromosome counts, in which the chromosome number of 2*n* = 61/62 (♀/♂) was lower by one in females (Figure 2c,d). CGH on female pachytene chromosomes showed no differentiated regions in the genome, and a single Z univalent was observed (Figure 2e–h). No differentiation nor the Z univalent was found in males (Figure 2i–l).

### 3.2. Chiasmia Clathrata (Ennominae)

**Figure 3:**
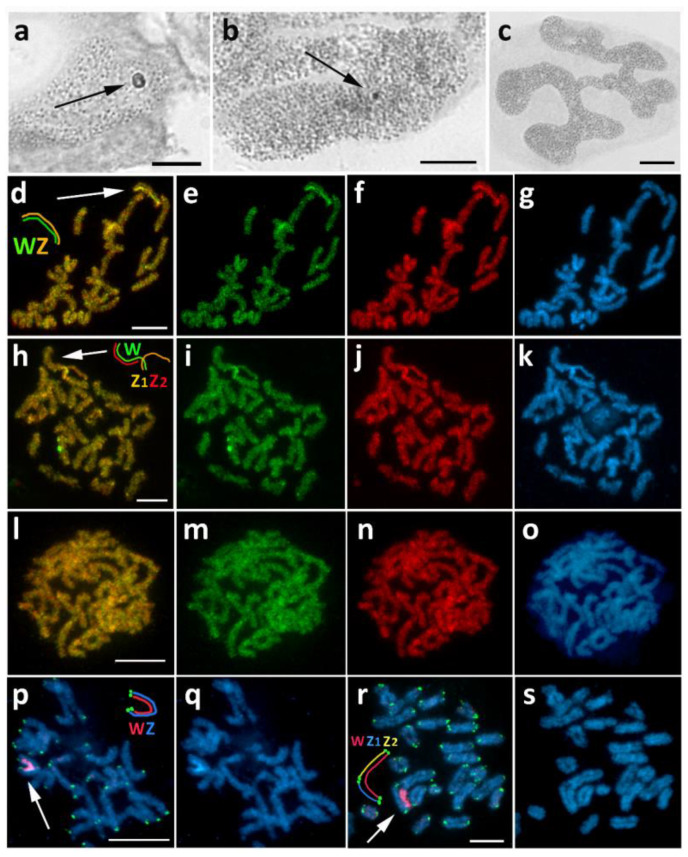
Sex chromosome systems in *Chiasmia clathrata*. (**a**–**c**) Polyploid nuclei stained with orcein showing a variable sex chromatin pattern in females from different broods, either normal (**a**) or miniature body (**b**), whereas it is absent in males (**c**). (**d–o**) Comparative genomic hybridization (CGH) on pachytene chromosomes revealed a WZ sex chromosome bivalent ((**d**), arrow and scheme) in females with normal sex chromatin (**d–g**) and a WZ1Z2 trivalent (**h**, arrow and scheme) in females with scattered sex chromatin (**h**–**k**); no chromosome was differentiated by CGH in males (**l–o**). Panels (**d**,**h**,**l**)—merged pictures of both probes; (**e**,**i**,**m**)—female genomic probe (green); (**f**,**j**,**n**)—male genomic probe (red); (**g**,**k**,**o**)—DAPI staining (light blue). (**p**–**s**) Fluorescence in situ hybridization (FISH) with W-painting probe (red) and (TTAGG)*_n_* telomeric probe (green) on pachytene chromosomes of females with the WZ bivalent (**p**,**q**) and females with the WZ_1_Z_2_ trivalent (**r**,**s**). Panels (**p**,**r**)—merged pictures of both probes; (**q**,**s**)—DAPI staining (light blue). In the WZ bivalent, the W-painting probe labeled the entire W chromosome (**p**, arrow and scheme). In the WZ_1_Z_2_ trivalent, less than half of the W chromosome was labeled with the W-painting probe, and telomeric signals confirmed the presence of two Z chromosomes (**r**, arrow and scheme). Bar = 10 µm.

In *C. clathrata*, we detected variability in the sex chromatin appearance. Female offspring in some broods showed normal sex chromatin (Figure 3a), while in other broods, they showed miniature sex chromatin bodies (Figure 3b). No sex chromatin was found in males (Figure 3c). The following cytogenetic analysis using CGH distinguished two types of sex chromosome systems: in broods with normal sex chromatin, we found the classical WZ system (Figure 3d–g), while in broods with miniature or scattered sex chromatin we found a WZ_1_Z_2_ system (Figure 3h–k). In both cases, the W chromosomes were not composed of conspicuous heterochromatin and were identified by both genomic probes, with the female probe signals being slightly stronger. They were mostly indiscernible by DAPI staining, although when observed in the pachytene nuclei of nurse cells, they were occasionally highlighted with DAPI, probably due to higher chromosome condensation. The existence of two sex chromosome systems was confirmed by FISH with the telomeric probe combined with the W-painting probe, which was prepared from microdissected sex chromatin bodies of WZ females. In females with normal sex chromatin, the W-painting probe labeled the W chromosome along its entire length and the telomeric probe hybridized to the ends of the WZ bivalent (Figure 3p,q). However, in females from two broods with fragmented sex chromatin, only part of the W chromosome was labeled with the W-painting probe, and telomeric signals were detected at both ends of this large W chromosome as well as at both ends of its two pairing partners, Z_1_ and Z_2_ (Figure 3r,s). These results strongly suggest that the ancestral W chromosome underwent fusion with an autosome, forming a neo-W chromosome. The homologous autosome thus became the Z_2_ sex chromosome. No chromosome was differentiated by CGH in male pachytene complements (Figure 3l–o). The exact chromosome number could not be identified due to an insufficient number of suitable-quality mitotic metaphases.

### 3.3. Epirrhoe Alternata (Larentiinae)

**Figure 4:**
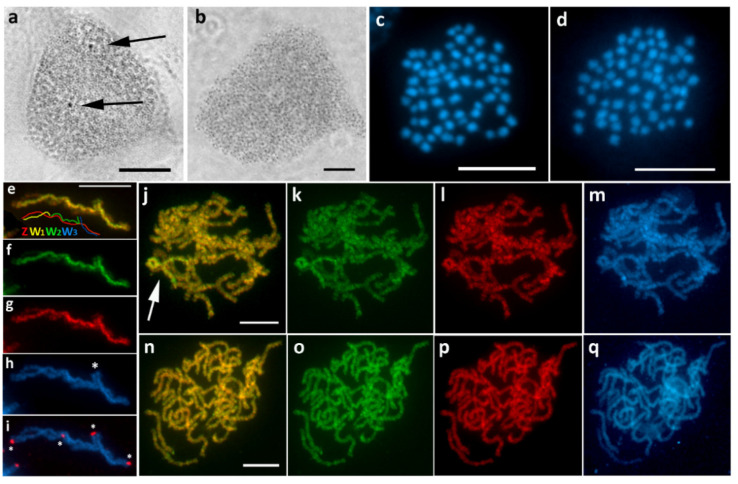
Sex chromosome multivalent in *Epirrhoe alternata*. (**a**,**b**) Polyploid nuclei stained with orcein showing miniature dot-like sex chromatin bodies in females ((**a**), arrows) but not in males (**b**). (**c**,**d**) Mitotic metaphase chromosomes stained with DAPI showing 2*n* = 64 in females (**c**) and 2*n* = 62 in males (**d**). (**e**–**h**) A sex chromosome quadrivalent, W_1_W_2_W_3_Z, revealed by CGH (**e**, scheme) and confirmed by reprobing using FISH with telomeric probe ((**i**), asterisks); note a pair of telomeric signals colocalized with two DAPI-positive heterochromatin blocks at the ends of W_2_ and W_3_ ((**h**), asterisk). (**j**–**m**) CGH on female pachytene chromosomes showing the sex chromosome multivalent highlighted with both genomic probes, with female signal being slightly stronger ((**j**), arrow). (**n**–**q**) CGH on male pachytene chromosomes without any differentiated regions. Panels (**e**,**j**,**n**)—merged pictures of both genomic probes; (**f**,**k**,**o**)—female genomic probe (green); (**g**,**l**,**p**)—male genomic probe (red); (**h**,**m**,**q**)—DAPI staining (light blue); (**i**)—merged picture of DAPI staining and (TTAGG)*_n_* telomeric probe (red). Bar = 10 µm.

Females of *E. alternata* showed scattered sex chromatin with a variable appearance, often forming either one or two miniature heterochromatic bodies, occasionally even smaller dot-like bodies (Figure 4a). No sex chromatin was found in males (Figure 4b). In all females examined, CGH analysis uncovered a sex chromosome multivalent consisting of differentiated W chromosomes, highlighted by both genomic probes with the female signal being slightly stronger (Figure 4j–m), which was not seen in males (Figure 4n–q). Since it was necessary to distinguish between the individual chromosomes of the multivalent, the CGH slides were reprobed by FISH with a telomeric probe (Figure 4e–i). In addition to standard telomeric signals at the ends of the multivalent, we detected two additional pairs of hybridization signals (Figure 4i). One of them also colocalized with DAPI-positive heterochromatin blocks, which are located terminally on the respective W chromosomes (Figure 4h,i). Taken together, these results suggest the presence of a sex chromosome quadrivalent consisting of three separate W chromosomes paired with a single Z chromosome (Figure 4e). Therefore, the sex chromosome constitution in this species is W_1_W_2_W_3_Z/ZZ (♀/♂). Consistent with this finding, the diploid chromosome number was determined to be 2*n* = 64 in females and 2*n* = 62 in males (Figure 4c,d).

### 3.4. Hylaea Fasciaria (Ennominae)

**Figure 5:**
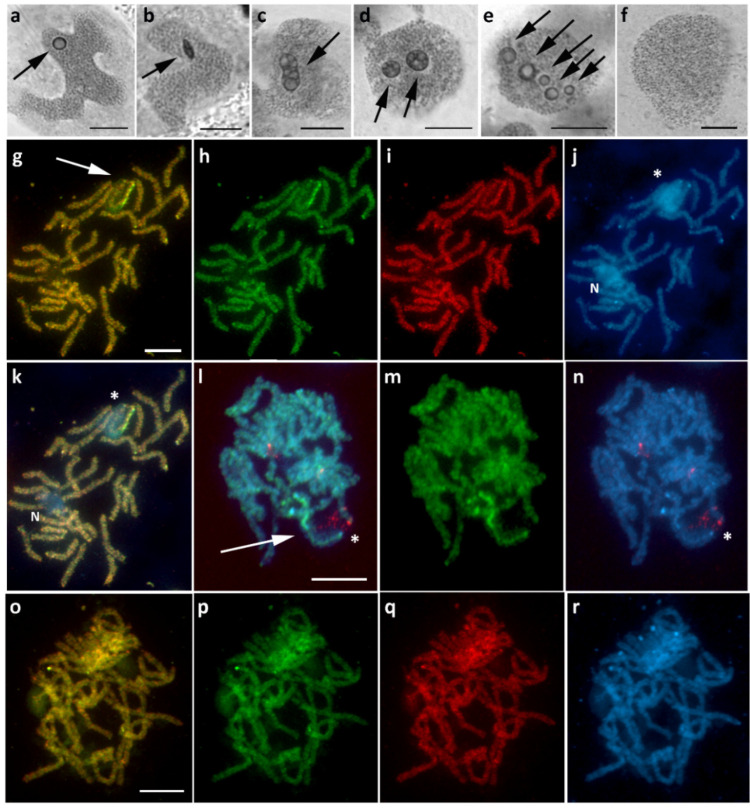
Multiple sex chromatin bodies and nucleolus-associated W chromosome in *Hylaea fasciaria*. (**a**–**f**) Polyploid nuclei stained with orcein showing variable forms of sex chromatin in females (**a**–**e**, arrows), from a regular single body or deformed body to multiple bodies, while it was absent in males (**f**). (**g**–**k**) CGH on female pachytene chromosomes identified a WZ bivalent ((**g**), arrow), in which the strongly heterochromatinized part of the W chromosome was preferentially labeled with the female genomic probe (**g**–**i**), and the other part of the WZ bivalent was associated with the nucleolus, well visible after DAPI staining ((**j**,**k**); asterisk); note the second nucleolus (N) in the same pachytene complement (**j**,**k**). (**l–n**) GISH combined with FISH with 18S rDNA probe on female pachytene chromosomes identified the WZ bivalent ((**l**), arrow) according to the W chromosome labeled by the female genomic probe (**l**,**m**) and the associated nucleolus by scattered signals of the 18S rDNA probe (**l**,**n**; red signals, asterisk). (**o**–**r**) CGH on male pachytene chromosomes without any differentiated region. Panels (**g**,**o**)—merged pictures of both genomic probes; (**h**,**m**,**p**)—female genomic probe (green); (**i**,**q**)—male genomic probe (red); (**j**,**r**)—DAPI staining (light blue); (**k**)—merged picture of both genomic probes and DAPI staining; (**l**)—merged picture of the female genomic probe, 18S rDNA probe, and DAPI staining; (**n**)—merged picture of 18S rDNA probe and DAPI staining. Bar = 10 µm.

Sex chromatin in *H. fasciaria* females was rather unusual. Apart from a single spherical conspicuous body (Figure 5a), we also observed two or more bodies within one polyploid nucleus, often differing in size and/or shape (Figure 5d,e); occasionally, we found a single oval-shaped or otherwise deformed body (Figure 5b,c). No sex chromatin was found in males (Figure 5f). These findings were partially elucidated by the CGH results, as the W chromosome was preferentially labeled with the female genomic probe and composed of two parts: a strongly heterochromatic part and a euchromatic-like part (Figure 5g–k). Interestingly, the latter part of the WZ bivalent was associated with the nucleolus (Figure 5j,k). Application of the 18S rDNA probe combined with GISH detected many scattered, disorganized signals throughout the nucleolus region (Figure 5l–n). We assume that this is due to loose chromatin loops, suggesting continuous high transcriptional activity in the nucleolar organizer region (NOR). In males, no differentiated chromosomes were identified (Figure 5o–r). Due to the lack of material, it was not possible to determine the number of chromosomes in this species.

### 3.5. Hypomecis Atomaria (Ennominae)

**Figure 6:**
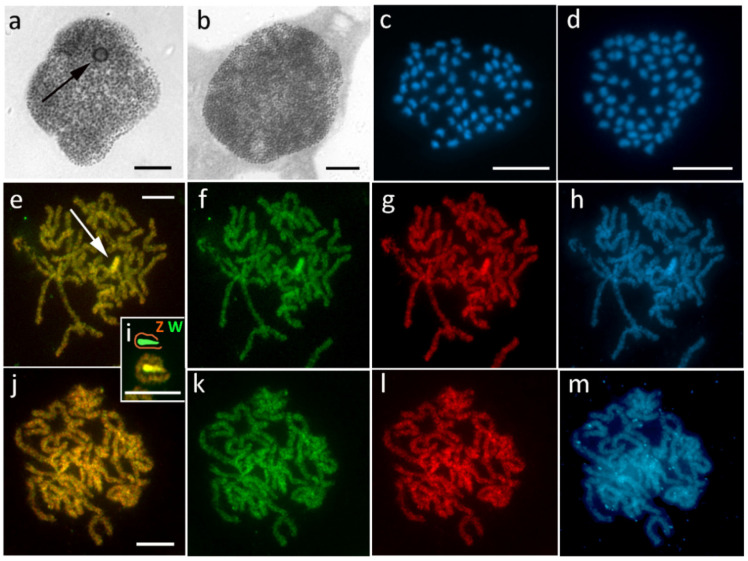
Highly differentiated WZ sex chromosomes in *Hypomecis atomaria*. (**a**,**b**) Polyploid nuclei stained with orcein showing a voluminous sex chromatin body in females ((**a**), arrow) and missing sex chromatin in males (**b**). (**c**,**d**) Mitotic metaphase chromosomes stained with DAPI showing 2*n* = 62 in both females (**c**) and males (**d**). (**e**–**m**) Comparative genomic hybridization (CGH) on pachytene chromosomes identified in females (**e**–**i**) a highly differentiated W chromosome ((**e**), arrow), composed of prominent DAPI-positive heterochromatin (**h**). In the WZ bivalent, the W chromosome formed a short, thick rod surrounded by a long Z chromosome ((**i**), scheme). In males, no chromosome was differentiated by CGH (**j**–**m**). Panels (**e**,**i**,**j**)—merged pictures of both probes; (**f**,**k**)—female genomic probe (green); (**g**,**l**)—male genomic probe (red); (**h**,**m**)—DAPI staining (light blue). Bar = 10 µm.

*Hypomecis atomaria* (previously *Ematurga atomaria*, see the work of [51] for the nomenclatural change) was used as our first control species. In highly polyploid nuclei, sex chromatin formed a typical, roundish and conspicuous body in females, but not in males (Figure 6a,b). The diploid number of chromosomes was the same in both sexes, 2*n* = 62 (Figure 6c,d), suggesting a WZ/ZZ sex chromosome system. Using CGH, we identified a curious WZ bivalent in female pachytene complements (Figure 6e–h), with a remarkably small, fully heterochromatic W chromosome that was often completely surrounded by the Z chromosome (Figure 6i). Since both genomic probes hybridized evenly to the W chromosome (Figure 6f,g), we assume that it is predominantly composed of repetitive DNA sequences common to both sexes. In males, no chromosome was differentiated by CGH (Figure 6j–m).

### 3.6. Operophtera Brumata (Larentiinae)

**Figure 7:**
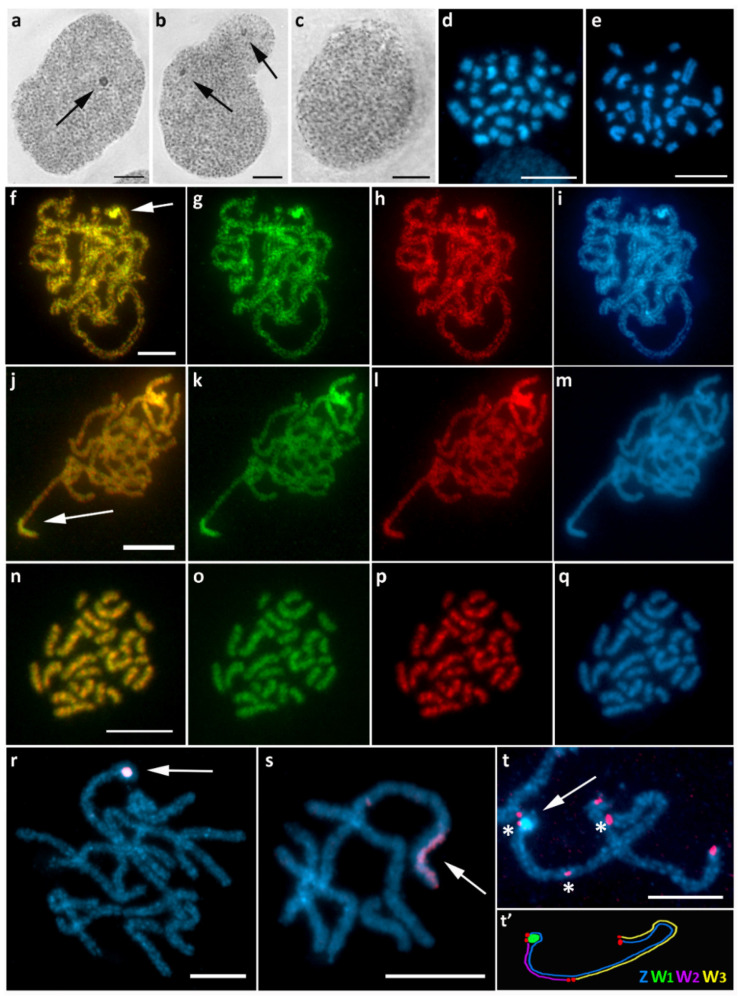
Sex chromosome multivalent and reduced chromosome number in *Operophtera brumata*. (**a**–**c**) Polyploid nuclei stained with orcein showing a single conspicuous sex chromatin body ((**a**), arrow) and occasionally two bodies ((**b**), arrows) in females, but no sex chromatin in males (**c**). (**d**,**e**) Mitotic chromosomes stained with DAPI showing 2*n* = 30 in females (**d**) and 2*n* = 28 in males (**e**). (**f**–**m**) CGH on female pachytene chromosomes identified a terminal W chromosome segment, either forming a heterochromatin body (**f**–**i**, arrow) or a short region paired with the Z chromosome (**j**–**m**, arrow), highly differentiated with the female genomic probe, but also highlighted with the male genomic probe. (**n**–**q**) No differentiated regions were found after CGH on male mitotic chromosomes. (**r**,**s**) FISH with the W_1_-painting probe showing two forms of the W_1_ chromosome, condensed (**r**, arrow) and stretched ((**s**), arrow). (**t**,**t’**) FISH with the (TTAGG)*_n_* telomeric probe revealed a sex chromosome quadrivalent consisting of a fully differentiated W_1_ chromosome ((**t**), arrow) and two undifferentiated sex chromosomes, W_2_ and W_3_, separated by telomeric signals ((**t**), asterisks), as shown in the scheme (**t’**). Panels (**f**,**j**,**n**)—merged pictures of both genomic probes; (**g**,**k**,**o**)—female genomic probe (green); (**h**,**l**,**p**)—male genomic probe (red); (**i**,**m**,**q**)—DAPI staining (light blue); (**r**,**s**)—merged picture of W_1_-painting probe (red) and DAPI; (**t**)—merged picture of (TTAGG)*_n_* telomeric probe (red) and DAPI. Bar = 10 µm.

In *O. brumata*, a well-visible sex chromatin body (eventually two bodies) foreshadowed a highly differentiated W chromosome in females (Figure 7a,b), while no sex chromatin was found in males (Figure 7c). The diploid number of chromosomes was significantly reduced and differed between the sexes, with 2*n* = 30 in females and 2*n* = 28 in males; the individual chromosomes also varied greatly in size (Figure 7d,e), some of them being much larger than usual lepidopteran chromosomes. These two features combined indicate multiple fusions that formed the karyotype of this species. In pachytene oocytes, CGH strongly highlighted a relatively small chromosomal segment, the anticipated W chromosome (Figure 7f–m), which was not seen in pachytene spermatocytes (Figure 7n–q). Slightly preferential labeling with the female genomic probe was observed, although the male genomic probe also hybridized to the W chromosome (Figure 7g,h,k,l). This finding suggests the presence of female-specific sequences in combination with an abundance of common repetitive sequences on this chromosome. In most pachytene figures, the W chromosome was condensed into a compact roundish body (Figure 7f–i,r,t). Occasionally we could also observe it in a stretched form (Figure 7j–m,s). The peculiar shape of the heterochromatin part of W suggested that there could be multiple W chromosomes pairing with the Z chromosome. Thus, we performed reprobing by FISH with a telomeric probe to detect the ends of potential multiple sex chromosomes. Based on all of the results, we concluded that this highly degenerate heterochromatic chromosome is probably the ancestral W chromosome, W_1_ (Figure 7t). It was also strongly labeled with the painting probe prepared by microdissection of sex chromatin, thus confirming that sex chromatin consists only of W_1_ chromosome copies (Figure 7r,s). Given the different number of chromosomes between the sexes and the location of telomeres, we concluded that there are two other undifferentiated sex chromosomes, W_2_ and W_3_, pairing with a long Z chromosome (Figure 7t’). Since the chromosome number in *O. brumata* is much lower than the ancestral lepidopteran number (2*n* = 62), and only one of the W chromosomes is heterochromatin-rich, we assume that the Z chromosome underwent fusions with two autosomes, forming the neo-Z chromosome. The homologs of the fused autosomes then became the euchromatin-rich W_2_ and W_3_.

### 3.7. Peribatodes Rhomboidaria (Ennominae)

**Figure 8:**
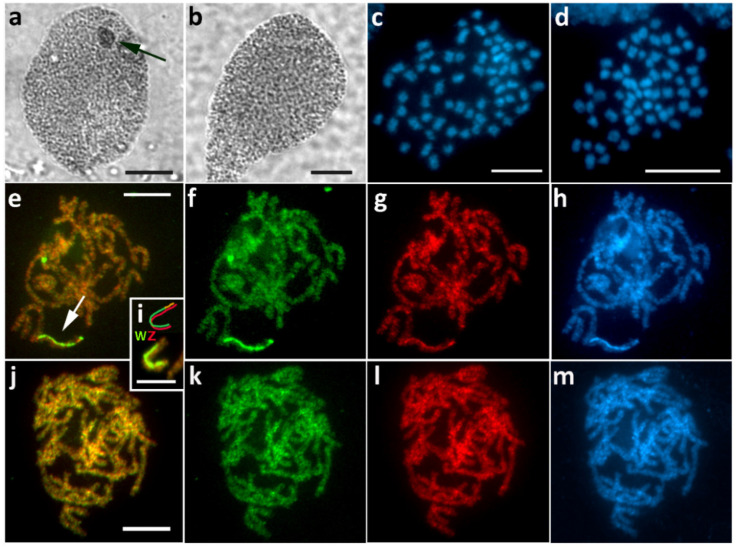
WZ sex chromosomes of *Peribatodes rhomboidaria* with W enriched in female-specific sequences. (**a**,**b**) Polyploid nuclei stained with orcein showing conspicuous sex chromatin in females (**a**) but not in males (**b**). (**c**,**d**) Mitotic metaphase chromosomes stained with DAPI showing 2*n* = 62 in both females (**c**) and males (**d**). (**e**–**i**) CGH on female pachytene chromosomes identified a WZ bivalent with a well-differentiated, DAPI-positive W chromosome, strongly labeled by the female genomic probe except for the terminal region ((**e**), arrow; (**i**) and scheme). (**j**–**m**) CGH on male pachytene chromosomes without any differentiated region. Panels (**e**,**i**,**j**)—merged pictures of both genomic probes; (**f**,**k**)—female genomic probe (green); (**g**,**l**)—male genomic probe (red); (**h**,**m**)—DAPI staining (light blue). Bar = 10 µm.

*Peribatodes rhomboidaria* was used as our second control species due to the typical, conspicuous sex chromatin body found in females but not in males (Figure 8a,b) and also due to the ancestral number of chromosomes in both sexes, 2*n* = 62 (Figure 8c,d). Accordingly, CGH revealed a highly differentiated, DAPI-positive W chromosome in females, preferentially labeled with the female genomic probe along almost the entire length of the chromosome, except for one terminal region that resembled the Z chromosome and autosomes (Figure 8e–i). No differentiated chromosome was found in males (Figure 8j–m).

### 3.8. Pseudopanthera Macularia (Ennominae)

**Figure 9:**
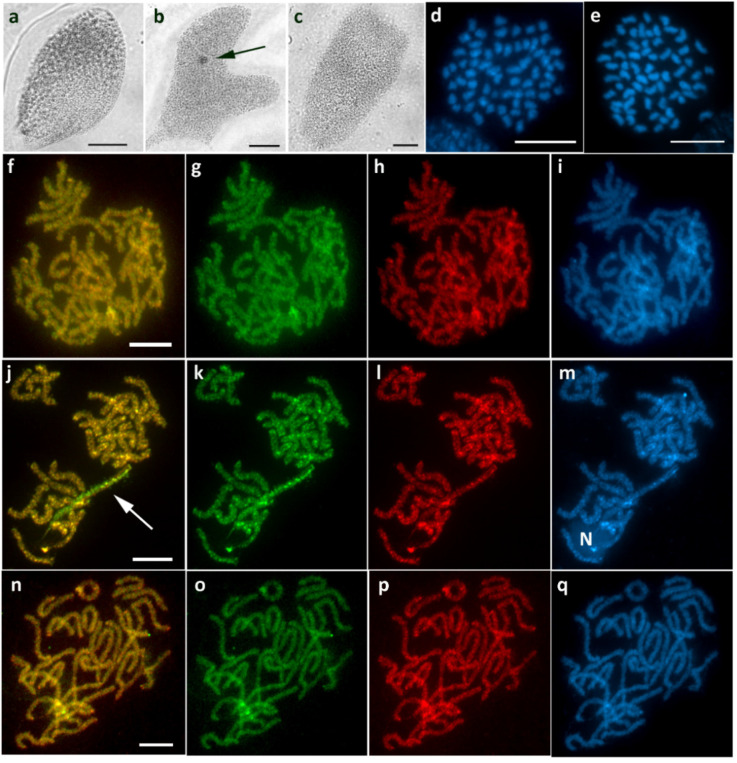
WZ sex chromosomes with variable W in *Pseudopanthera macularia*. (**a**–**c**) Orcein-stained polyploid nuclei showing the absence of sex chromatin in females from the first brood (**a**), a small sex chromatin body in females from another brood (**b**), and no sex chromatin in males (**c**). (**d**,**e**) Mitotic metaphase chromosomes stained with DAPI showing 2*n* = 62 in both females (**d**) and males (**e**). (**f**–**m**) CGH on female pachytene chromosomes failed to identify the WZ bivalent, suggesting the presence of an undifferentiated W chromosome in the first brood (**f**–**i**), whereas, in two other broods, the W chromosome was strongly labeled with the female genomic probe ((**j**–**m**), arrow); note nucleolus (N). (**n**–**q**) CGH on male pachytene chromosomes without any differentiated chromosome. Panels (**f**,**j**,**n**)—merged pictures of both probes; (**g**,**k**,**o**)—female genomic probe (green); (**h**,**l**,**p**)—male genomic probe (red); (**i**,**m**,**q**)—DAPI staining (light blue). Bar = 10 µm.

Intraspecific variability was observed in *P. macularia*. In the first brood, sex chromatin was absent in female progeny (Figure 9a), and CGH did not reveal any differentiated chromosome (Figure 9f–i). Such findings would indicate the absence of a W chromosome; however, since the chromosome number was the same 2*n* = 62 in both sexes (Figure 9d,e) and no Z univalent was found in females, we assume a WZ sex chromosome system with an undifferentiated W chromosome. In the other two examined broods, we found a relatively small sex chromatin body in females (Figure 9b), and CGH identified a WZ bivalent with a W chromosome preferentially labeled with the female genomic probe (Figure 9j–m). We did not detect any sex chromatin (Figure 9c) or any differentiated chromosome by CGH in all examined males (Figure 9n–q).

To summarize, we detected various forms of W chromosomes in females of all species studied, except *A. punctulata*, in which a Z univalent was found. As expected, we did not find any W chromosomes in males. We also determined chromosome numbers when possible. Most species showed a diploid number of 2*n* = 62, which corresponds to the ancestral number of chromosomes in Lepidoptera [20,25]. However, in *A. punctulata*, *E. alternata*, and *O. brumata*, there were differences between sexes, indicating either the W chromosome absence or the existence of multiple sex chromosomes. Moreover, the chromosome number in *O. brumata* was significantly reduced. The results obtained are summarized in Table 1.

**Table 1:** Overview of results.

Species	2n ♀/♂	SC ♀	W Composition + Features	Sex Chromatin
*A. punctulata*	61/62	Z0	absent (Z univalent recorded)	absent
*C. clathrata*	n.d.	WZ	female enriched/common repetitive (slightly DAPI+)	normal
	n.d.	WZ_1_Z_2_	neo-W with 2 parts: female enriched/common repetitive (slightly DAPI+) and undifferentiated	scattered
*E. alternata*	64/62	W_1_W_2_W_3_Z	common repeats/female enriched, DAPI+ blocks on W_2_, W_3_	scattered
*H. fasciaria*	n.d.	WZ	2 parts: female enriched (DAPI+) and undifferentiated (nucleolus-associated)	normal/multiple
*H. atomaria*	62/62	WZ	common repeats, DAPI+, small size	normal
*O. brumata*	30/28	W_1_W_2_W_3_Z	W_1_ female enriched/common repeats, DAPI+, W_2_ and W_3_ undifferentiated; neo-Z	normal/double
*P. rhomboidaria*	62/62	WZ	female enriched, DAPI+	normal
*P. macularia*	62/62	WZ	female enriched	normal
			undifferentiated	absent

2n **♀/♂**, diploid chromosome number in females/males. SC **♀,** sex chromosome constitution in females. W composition, prevalence of certain types of sequences determined by CGH (female enriched versus common repetitive sequences). DAPI+, DAPI-positive heterochromatin. n.d., not determined.

## 4. Discussion

In this work, we performed an extensive sex chromatin survey in 50 species of the family Geometridae. While females of most species displayed a single, normal-looking sex chromatin body, various exceptions were found, including miniature or scattered bodies, multiple bodies, or the complete absence of sex chromatin. Subsequent cytogenetic analysis of eight selected species, representing different types of sex chromatin, revealed a wide spectrum of W chromosome variants (including its absence), ranging from non-differentiated to fully degenerate W chromosomes, differing in number, size, and molecular composition. In addition, two cases of intraspecific W chromosome polymorphisms were recorded.

Our results, combined with the available literature, suggest a link between the sex chromatin presence and appearance and the constitution of sex chromosomes, specifically the W chromosome(s). In particular, the large conspicuous sex chromatin body (i.e., normal sex chromatin pattern) correlates with a high level of differentiation and consequent heterochromatinization of the W chromosome, which also often makes it easily recognizable after DAPI staining, such as in *E. kuehniella* [29], *C. pomonella* [46], *P. rhomboidaria*, and *H. atomaria* (this study). The correlation between the heterochromatin-rich W chromosome and the normal sex chromatin body occurs regardless of the actual W chromosome size. For example, the large W in *P. rhomboidaria* (this study) and small degenerated Ws in *H. atomaria* (this study) and in *Bicyclus* *anynana* [52] display similar sex chromatin bodies.

Hybridization of the W-painting probe in *O. brumata* further supports the opinion that especially the heterochromatin-rich W chromosome forms a typical sex chromatin body. In females of this species, we found a W_1_W_2_W_3_neo-Z sex chromosome constitution. We presume that W_1_ is most probably the ancestral highly degenerate heterochromatin-rich W chromosome, while W_2_ and W_3_ are of autosomal origin and consist of euchromatin. The W-painting probe prepared from microdissected sex chromatin in this species hybridized exclusively to the degenerated W_1_ chromosome. Therefore, the other two W chromosomes, which consist of euchromatin and resemble autosomes, do not form sex chromatin, indicating that it is the heterochromatin nature, not the presence of the W chromosome, which is vital for the sex chromatin formation.

Deviations from the typical appearance of sex chromatin can have several causes. For instance, we observed a variable occurrence of miniature or small sex chromatin bodies in *E. alternata*, although sex chromosome analysis showed the same results in all broods studied, i.e., a W_1_W_2_W_3_Z system in females. Although DAPI staining failed to clearly identify the W chromosomes, CGH reliably detected all three of them. The fact that the ancestral chromosome number of 2*n* = 62 is preserved in males of this species, while it is increased by 2 in females (2*n* = 64), suggests the origin of 3 Ws by fission of the ancestral W chromosome. However, a more complex origin involving fusions of sex chromosomes with autosomes and subsequent fissions, as demonstrated in *Leptidea* butterflies [11], cannot be ruled out without further research.

The sex chromatin status can also be influenced by an intraspecific polymorphism in the W chromosome composition. In Lepidoptera, intraspecific sex chromosome polymorphism has so far been found in *Samia cynthia* ssp. and *Orgyia thyellina* [8], *Danaus chrysippus* ssp. [53], and *C. clathrata* and *P. macularia* (this study). In the latter species, we were able to detect the W chromosome by CGH in females with regular sex chromatin. However, in females with a miniature or disintegrated sex chromatin, we failed to differentiate the W chromosome by CGH and thus to identify a WZ bivalent. Hence, the WZ/ZZ sex chromosome system was only deduced due to the same number of chromosomes in both sexes, 2*n* = 62. Because each W chromosome is inherited independently only in the female lineage and without meiotic recombination, we suggest that the differentiated and undifferentiated W chromosomes may have diverged by the acquisition of different types of sequences.

Variability of the W chromosome in *P. macularia* with consequent variability in sex chromatin presence shows that it is the particular sequence composition of the W in respective species, populations, or females that will or will not lead to the formation of normal sex chromatin. Impaired heterochromatinization might have various causes, including the lack of “booster” sequences (e.g., LINE elements) promoting the spread of a silencing factor [54]. Considering the high sex chromosome variability within Lepidoptera, individual sex chromosomes may or may not foster the silencing signals sufficiently. Additionally, the mere existence of heterochromatin on the sex chromosome does not automatically lead to the formation of the sex chromatin body. Thus, higher-order heterochromatin organization is required, such as changes in the phosphorylation of heterochromatin proteins, as seen in protozoan *Tetrahymena* [55]. In Lepidoptera, however, such molecular mechanism remains elusive.

Sex chromosome ability to establish and maintain heterochromatin on any level may be further impaired by translocation of a euchromatic (e.g., autosomal) segment. In *C. clathrata*, we observed two distinct forms of sex chromatin: (i) normal sex chromatin corresponding to the WZ system and (ii) miniature or scattered sex chromatin in females with the neo-WZ_1_Z_2_ system. In both cases, the ancestral W chromosome or its part in the neo-W chromosome was differentiated using CGH or FISH with the W-painting probe. In neo-WZ_1_Z_2_ females, the formation of normal sex chromatin was probably disrupted by the undifferentiated, autosome-derived part of the neo-W chromosome. Loss of the sex chromatin was already observed in natural species with a neo-W chromosome, such as the clouded Apollo, *Parnassius mnemosyne* [9], as well as in structural mutants of the W chromosome in *E. kuehniella* [18,29]. These authors suggested that the disruption of sex chromatin is caused by the tendency of transcriptionally active autosomal chromatin to disperse. The sex chromatin presence and appearance also depend on the size of translocated fragments, as shown in the T(W;Z) mutant lines of *E. kuehniella* [18], and on the specific sequence content. On the other hand, the formation of neo-W chromosomes does not always lead to the absence of sex chromatin. For example, the monarch butterfly, *Danaus plexippus*, has a neo-W chromosome with two clearly differentiated parts (old heterochromatic and new euchromatic) but still shows a single-sex chromatin body in female polyploid cells [56].

The euchromatic part of the W chromosome is probably also responsible for the formation of multiple sex chromatin bodies observed in *H. fasciaria* females (this study). However, unlike *C. clathrata*, these sex chromatin bodies were conspicuous and only had different sizes and shapes. In this species, we encountered an intriguing phenomenon. The WZ bivalent appeared to be immersed into the nucleolus, so the presence of a large cluster of genes for ribosomal RNA was expected. Yet, FISH with the 18S rDNA probe failed to localize such a cluster directly on the W and/or Z chromosomes. Instead, we detected scattered signals nearby the bivalent, possibly in loosen chromatin loops. Nevertheless, we suggest that the unusual appearance and number of sex chromatin bodies may be a side effect of the W chromosome association with the nucleolus and its potential link to high transcriptional activity in this region. Moreover, since the other part of the W in *H. fasciaria* is fully differentiated and heterochromatic, it is also possible that this chromosome is, in fact, a neo-W that arose by fusion between the ancestral W and an NOR-bearing autosome. Further research is needed to verify this option.

Finally, the absence of sex chromatin may in some cases truly indicate the absence of the W chromosome, as shown in *A. punctulata* (this study) or, e.g., in the common clothes moth, *Tineola bisselliella* [17], and three species of bagworms (Psychidae) [27]. The loss of the W chromosome in some species points to the dispensability of this chromosome and indicates the presence of yet unknown molecular mechanism of sex determination [57], different from the lepidopteran model species, *B. mori*, in which the W chromosome carries a sex-determining factor [58]. On the contrary, in the sex chromatin absence in one of *P. macularia* broods was probably caused by the low level of differentiation of the W chromosome (see above). This finding suggests that the actual number of lepidopteran species lacking the W chromosome, estimated by the sex chromatin absence (see the work of [24]), is, in fact, lower. As follows from this study, a detailed analysis of karyotype and sex chromosomes using methods of molecular cytogenetics is crucial to determine the presence or absence of the W chromosome. Moreover, multiple females of each species, preferably from more than one population, should be examined for possible intraspecific polymorphisms or anomalies. Such a thorough cytogenetic investigation may also lead to the discovery of derived sex chromosome systems, thus contributing to the understanding of the evolution of sex chromosomes in Lepidoptera (e.g., the work of [12,56,59,60,61]).

## 5. Conclusions

Our results and literature data suggest that there are two main factors influencing the sex chromatin formation: (i) the actual level of differentiation or degeneration of the W chromosome and (ii) the presence of a euchromatin segment on the W chromosome with potential transcriptional activity, usually acquired by structural rearrangements with autosomes or the Z chromosome. Our work revealed the astonishing variability of sex chromosomes in Geometridae species, emphasized the need to inspect sex chromosomes in more populations because of possible intraspecific polymorphisms, and showed that the sex chromatin assay is an easy tool to pinpoint species with atypical sex chromosomes. These species can then be studied at more detailed genomic and transcriptomic levels to unveil the molecular mechanisms of speciation, adaptation, sex determination, and sex chromatin formation.

## Supplementary Materials

The following are available online at https://www.mdpi.com/article/10.3390/cells10092230/s1, Table S1: Overview of sex chromatin occurrence in Geometridae, Table S2: List of species selected for detailed cytogenetic study.

## References

[1] Charlesworth D., Charlesworth B., Marais G. (2005). Steps in the evolution of heteromorphic sex chromosomes. J. Hered..

[2] Abbott J.K., Nordén A.K., Hansson B. (2017). Sex chromosome evolution: Historical insights and future perspectives. Proc. Biol. Sci..

[3] Charlesworth B. (1996). The evolution of chromosomal sex determination and dosage compensation. Curr. Biol..

[4] Furman B.L.S., Metzger D.C.H., Darolti I., Wright A.E., Sandkam B.A., Almeida P., Shu J.J., Mank J.E. (2020). Sex chromosome evolution: So many exceptions to the rules. Genome Biol. Evol..

[5] Nokkala S., Grozeva S., Kuznetsova V., Maryanska-Nadachowska A. (2003). The origin of the achiasmatic XY sex chromosome system in *Cacopsylla peregrina* (Frst.) (Psylloidea, Homoptera). Genetica.

[6] Clark F.E., Kocher T.D. (2019). Changing sex for selfish gain: B chromosomes of Lake Malawi cichlid fish. Sci. Rep..

[7] Traut W., Marec F. (1997). Sex chromosome differentiation in some species of Lepidoptera (Insecta). Chromosome Res..

[8] Yoshido A., Marec F., Sahara K. (2005). Resolution of sex chromosome constitution by genomic in situ hybridization and fluorescence in situ hybridization with (TTAGG)*_n_* telomeric probe in some species of Lepidoptera. Chromosoma.

[9] Vlašánek P., Bartoňová A., Marec F., Konvička M. (2017). Elusive *Parnassius mnemosyne* (Linnaeus, 1758) larvae: Habitat selection, sex determination and sex ratio (Lepidoptera: Papilionidae). SHILAP Rev. Lepidopt..

[10] Grützner F., Deakin J., Rens W., El-Mogharbel N., Marshall Graves J. (2003). The monotreme genome: A patchwork of reptile, mammal and unique features?. Comp. Biochem. Physiol. A Mol. Integr. Physiol..

[11] Yoshido A., Šíchová J., Pospíšilová K., Nguyen P., Šafář J., Provazník J., Voleníková A., Vila R., Marec F. (2020). Evolution of multiple sex-chromosomes associated with dynamic genome reshuffling in *Leptidea* wood-white butterflies. Heredity.

[12] Šíchová J., Ohno M., Dincă V., Watanabe M., Sahara K., Marec F. (2016). Fissions, fusions, and translocations shaped the karyotype and multiple sex chromosome constitution of the northeast-Asian wood white butterfly, *Leptidea amurensis*. Biol. J. Linn. Soc..

[13] Zrzavá M., Hladová I., Dalíková M., Šíchová J., Õunap E., Kubíčková S., Marec F. (2018). Sex chromosomes of the iconic moth *Abraxas grossulariata* (Lepidoptera, Geometridae) and its congener *A. sylvata*. Genes.

[14] Chirino M.G., Fourastie M.F., Cemteno N.D., Bressa M.J. (2020). Unusual chromosome polymorphism and heterochromatin variation in the Argentinean population of the necrophagous fly *Lucilia sericata* (Diptera: Calliphoridae), comparison with other populations and evolutionary aspects. Eur. J. Entomol..

[15] Sahara K., Yoshido A., Traut W. (2012). Sex chromosome evolution in moths and butterflies. Chromosome Res..

[16] Wright A.E., Dean R., Zimmer F., Mank J.E. (2016). How to make a sex chromosome. Nat. Commun..

[17] Dalíková M., Zrzavá M., Hladová I., Nguyen P., Šonský I., Flegrová M., Kubíčková S., Voleníková A., Kawahara A.Y., Peters R.S. (2017). New insights into the evolution of the W chromosome in Lepidoptera. J. Hered..

[18] Marec F., Traut W. (1994). Sex chromosome pairing and sex chromatin bodies in W-Z translocation strains of *Ephestia kuehniella* (Lepidoptera). Genome.

[19] Beldade P., Saenko S.V., Pul N., Long A.D. (2009). A gene-based linkage map for *Bicyclus*
*anynana* butterflies allows for a comprehensive analysis of synteny with the lepidopteran reference genome. PLoS Genet..

[20] Van’t Hof A.E., Nguyen P., Dalíková M., Edmonds N., Marec F., Saccheri I.J. (2013). Linkage map of the peppered moth, *Biston betularia* (Lepidoptera, Geometridae): A model of industrial melanism. Heredity.

[21] Fraïsse C., Picard M.A.L., Vicoso B. (2017). The deep conservation of the Lepidoptera Z chromosome suggests a non-canonical origin of the W. Nat. Commun..

[22] Traut W., Sahara K., Marec F. (2007). Sex chromosomes and sex determination in Lepidoptera. Sex Dev..

[23] Traut W., Vogel H., Glöckner G., Hartmann E., Heckel D.G. (2013). High-throughput sequencing of a single chromosome: A moth W chromosome. Chromosome Res..

[24] Traut W., Marec F. (1996). Sex chromatin in Lepidoptera. Q. Rev. Biol..

[25] Lukhtanov V.A. (2000). Sex chromatin and sex chromosome systems in nonditrysian Lepidoptera (Insecta). J. Zool. Syst. Evol. Res..

[26] Šíchová J., Nguyen P., Dalíková M., Marec F. (2013). Chromosomal evolution in tortricid moths: Conserved karyotypes with diverged features. PLoS ONE.

[27] Hejníčková M., Koutecký P., Potocký P., Provazníková I., Voleníková A., Dalíková M., Visser S., Marec F., Zrzavá M. (2019). Absence of W chromosome in Psychidae moths and implications for the theory of sex chromosome evolution in Lepidoptera. Genes.

[28] Rathjens B. (1974). Zur Funktion des W-Chromatins bei *Ephestia kuehniella* (Lepidoptera). Isolierung und Charakterisierung von W-Chromatin-Mutanten. Chromosoma.

[29] Traut W., Weith A., Traut G. (1986). Structural mutants of the W chromosome in *Ephestia* (Insecta, Lepidoptera). Genetica.

[30] Makee H., Tafesh N. (2007). Sex chromatin body as a cytogenetic marker of W chromosome aberrations in *Cydia pomonella* females. Area-Wide Control of Insect Pests.

[31] Traut W., Clarke C.A. (1997). Karyotype evolution by chromosome fusion in the moth genus *Orgyia*. Hereditas.

[32] Fuková I., Traut W., Vítková M., Nguyen P., Kubíčková S., Marec F. (2007). Probing the W chromosome of the codling moth, *Cydia pomonella*, with sequences from microdissected sex chromatin. Chromosoma.

[33] Vítková M., Fuková I., Kubíčková S., Marec F. (2007). Molecular divergence of the W chromosomes in pyralid moths (Lepidoptera). Chromosome Res..

[34] Traut W., Sahara K., Otto T.D., Marec F. (1999). Molecular differentiation of sex chromosomes probed by comparative genomic hybridization. Chromosoma.

[35] Sahara K., Marec F., Eickhoff U., Traut W. (2003). Moth sex chromatin probed by comparative genomic hybridization (CGH). Genome.

[36] Green J.E., Dalíková M., Sahara K., Akam M., Marec F. (2016). XX/XY system of sex determination in the geophilomorph centipede *Strigamia maritima*. PLoS ONE.

[37] Sihvonen P., Mutanen M., Kaila L., Brehm G., Hausmann A., Staude H.S. (2011). Comprehensive molecular sampling yields a robust phylogeny for geometrid moths (Lepidoptera: Geometridae). PLoS ONE.

[38] Traut W., Mosbacher G.C. (1968). Geschlechtschromatin bei Lepidopteren. Chromosoma..

[39] Ennis T.J. (1976). Sex chromatin and chromosome numbers in Lepidoptera. Can. J. Genet. Cytol..

[40] Makino S. (1951). An Atlas of the Chromosome Numbers in Animals.

[41] Robinson R. (1970). Lepidoptera Genetics.

[42] Murillo-Ramos L., Brehm G., Sihvonen P., Hausmann A., Holm S., Reza Ghanavi H., Õunap E., Truuverk A., Staude H., Friedrich E. (2019). A comprehensive molecular phylogeny of Geometridae (Lepidoptera) with a focus on enigmatic small subfamilies. Peer J..

[43] Mediouni J., Fuková I., Frydrychová R., Dhouibi M.H., Marec F. (2004). Karyotype, sex chromatin and sex chromosome differentiation in the carob moth, *Ectomyelois ceratoniae* (Lepidoptera: Pyralidae). Caryologia.

[44] Winnepenninckx B., Backeljau T., de Wachter R. (1993). Extraction of high molecular weight DNA from molluscs. Trends Genet..

[45] Kato A., Albert P.S., Vega J.M., Birchler J.A. (2006). Sensitive fluorescence in situ hybridization signal detection in maize using directly labeled probes produced by high concentration DNA polymerase nick translation. Biotech. Histochem..

[46] Fuková I., Nguyen P., Marec F. (2005). Codling moth cytogenetics: Karyotype, chromosomal location of rDNA, and molecular differentiation of sex chromosomes. Genome.

[47] Okazaki S., Tsuchida K., Maekawa H., Ishikawa H., Fujiwara H. (1993). Identification of a pentanucleotide telomeric sequence, (TTAGG)*_n_*, in the silkworm *Bombyx mori* and in other insects. Mol. Cell. Biol..

[48] Ijdo J.W., Wells R.A., Baldini A., Reeders S.T. (1991). Improved telomere detection using a telomere repeat probe (TTAGGG)_n_ generated by PCR. Nucleic Acids Res..

[49] Sahara K., Marec F., Traut W. (1999). TTAGG telomeric repeats in chromosomes of some insects and other arthropods. Chromosome Res..

[50] Shibata F., Sahara K., Naito Y., Yasukochi Y. (2009). Reprobing multicolor FISH preparations in lepidopteran chromosome. Zool. Sci..

[51] Murillo-Ramos L., Chazot N., Sihvonen P., Õunap E., Jiang N., Han H., Clarke J.T., Davis R.B., Tammaru T., Wahlberg N. (2021). Molecular phylogeny, classification, biogeography and diversification patterns of a diverse group of moths (Geometridae: Boarmiini). Mol. Phylogenet. Evol..

[52] Van’t Hof A.E., Marec F., Saccheri I.J., Brakefield P.M., Zwaan B.J. (2008). Cytogenetic characterization and AFLP-based genetic linkage mapping for the butterfly *Bicyclus anynana*, covering all 28 karyotyped chromosomes. PLoS ONE.

[53] Smith D.A.S., Gordon I.J., Traut W., Herren J., Collins S., Martins D.J., Saitoti K., Ireri P., Ffrench-Constant R. (2016). A neo-W chromosome in a tropical butterfly links colour pattern, male-killing, and speciation. Proc. R. Soc. B.

[54] Loda A., Brandsma J.H., Vassilev I., Servant N., Loos F., Amirnasr A., Splinter E., Barillot E., Poot R.A., Heard E. (2017). Genetic and epigenetic features direct differential efficiency of Xist-mediated silencing at X-chromosomal and autosomal locations. Nat. Commun..

[55] Kataoka K., Noto T., Mochizuki K. (2015). Phosphorylation of an HP1-like protein regulates heterochromatin body assembly for DNA elimination. Dev. Cell..

[56] Mongue A.J., Nguyen P., Voleníková A., Walters J.R. (2017). Neo-sex chromosomes in the Monarch butterfly, *Danaus plexippus*. G3 Genes Genomes Genet..

[57] Yoshido A., Marec F., Sahara K. (2016). The fate of W chromosomes in hybrids between wild silkmoths, *Samia cynthia* ssp.: No role in sex determination and reproduction. Heredity.

[58] Kiuchi T., Koga H., Kawamoto M., Shoji K., Sakai H., Arai Y., Ishihara G., Kawaoka S., Sugano S., Shimada T. (2014). A single female-specific piRNA is the primary determiner of sex in the silkworm. Nature.

[59] Yoshido A., Sahara K., Marec F., Matsuda Y. (2011). Step-by-step evolution of neo-sex chromosomes in geographical populations of wild silkmoths, *Samia cynthia* ssp. Heredity.

[60] Carabajal Paladino L.Z., Provazníková I., Berger M., Bass C., Aratchige N.S., López S.N., Marec F., Nguyen P. (2019). Sex chromosome turnover in moths of the diverse superfamily Gelechioidea. Genome Biol. Evol..

[61] Šíchová J., Voleníková A., Dincă V., Nguyen P., Vila R., Sahara K., Marec F. (2015). Dynamic karyotype evolution and unique sex determination systems in *Leptidea* wood white butterflies. BMC Evol. Biol..

