# Supplementary material for: Degenerated, Undifferentiated, Rearranged, Lost: High Variability of Sex Chromosomes in Geometridae (Lepidoptera) Identified by Sex Chromatin
Source: Cells. 2021 Aug 28;10(9):2230. doi: 10.3390/cells10092230 (PMC8468057; doi:10.3390/cells10092230)
Supplement: Supplementary file 1 [file cells-10-02230-s001.zip › Table_S2.pdf]

Species	Sex chromatin F/M *	Cytogenetics F/M **	Broods analysed ***	Localities of collection ****
Aethalura punctulata	8/6	5/6	2	České Budějovice CZ
Chiasmia clathrata	28/15	11/2	4	České Budějovice + Kuří (Benešov nad Černou) CZ
Epirrhoe alternata	25/9	19/5	5	České Budějovice + Kuří (Benešov nad Černou) + Vyšenské kopce (Český Krumlov) CZ + Karilatsi (Põlva) EE
Hylaea fasciaria	7/3	4/2	1	Karilatsi (Põlva) EE
Hypomecis atomaria	12/5	7/6	3	České Budějovice + Vyšenské kopce (Český Krumlov) CZ
Operophtera brumata	11/3	9/6	4	České Budějovice CZ
Peribatodes rhomboidaria	7/8	7/4	3	České Budějovice + Javorník (Hodonín) CZ
Pseudopanthera macularia	5/4	4/5	3	Brněnec (Svitavy) + Sulíkov (Blansko) + Chvalnov-Lísky (Kroměříž) CZ

* Total number of individuals (including adults) inspected for sex chromatin: females/males

** Total number of individuals in analysed/karyotyped by molecular cytogenetics (CGH, GISH, FISH, DAPI staining): females/males

*** Number of broods (i.e. offsprings of a single female) inspected by molecular cytogenetics

**** Localities of collection: CZ = Czech Republic; EE = Estonia
